# Efficacy of a new cancer treatment strategy based on eradication of tumor-initiating stem cells in a mouse model of Krebs-2 solid adenocarcinoma

**DOI:** 10.18632/oncotarget.25503

**Published:** 2018-06-19

**Authors:** Ekaterina A. Potter, Anastasia S. Proskurina, Genrikh S. Ritter, Evgenia V. Dolgova, Valeriy P. Nikolin, Nelly A. Popova, Oleg S. Taranov, Yaroslav R. Efremov, Sergey I. Bayborodin, Aleksandr A. Ostanin, Elena R. Chernykh, Nikolay A. Kolchanov, Sergey S. Bogachev

**Affiliations:** ^1^ Institute of Cytology and Genetics, Siberian Branch of the Russian Academy of Sciences, Novosibirsk, Russia; ^2^ Novosibirsk State University, Novosibirsk, Russia; ^3^ State Research Center of Virology and Biotechnology “Vector”, Koltsovo, Novosibirsk, Russia; ^4^ Research Institute of Fundamental and Clinical Immunology, Novosibirsk, Russia

**Keywords:** DNA internalization, synchronization, cyclophosphamide, seasonal cyclicity, repair cycle

## Abstract

Krebs-2 solid carcinoma was cured using a new “3+1” strategy for eradication of Krebs-2 tumor-initiating stem cells. This strategy was based on synchronization of these cells in a treatment-sensitive phase of the cell cycle. The synchronization mechanism, subsequent destruction of Krebs-2 tumor-initiating stem cells, and cure of mice from a solid graft were found to depend on the temporal profile of the interstrand cross-link repair cycle. Also, the temporal profile of the Krebs-2 interstrand repair cycle was found to have a pronounced seasonal cyclicity at the place of experiments (Novosibirsk, Russia). As a result, the therapeutic effect that is based on application of the described strategy, originally developed for the “winter repair cycle” (November−April), is completely eliminated in the summer period (June−September). We conclude that оne of the possible and the likeliest reasons for our failure to observe the therapeutic effects was the seasonal cyclicity in the duration of the interstrand repair cycle, the parameter that is central to our strategy.

## INTRODUCTION

In our recent publications, two new general biological phenomena have been discovered and characterized. The first is the ability of low-differentiated cells of different genesis to internalize fragments of extracellular double-stranded DNA using the natural internalization mechanism. The second is the ability of double-stranded DNA fragments delivered to internal compartments of tumor initiating stem cells (TISCs) to interfere with repair of interstrand cross-links (ICLs) induced by a cross-linking cytostatic. The exact molecular underpinnings of this interference remain elusive. Yet, several scenarios appear likely, for instance extracellular DNA may interfere with the processing of NER and homology repair DNA intermediates; alternatively, double-stranded DNA ends may exhaust the pool of repair machinery proteins or launch distinct repair cascades. Whichever the case may be, this interference leads to eradicate TISCs from the tumor site [1–8].

A study by Potter and co-workers [5] demonstrated that there is a regimen for the joint use of a cytostatic cyclophosphamide (CP) and a composite DNA-based preparation. It enables simultaneous elimination of committed tumor cells and eradication of TISCs from the tumor site. Eradication of TISCs thereby deprives the tumor of its tumorigenic potential. The residual non-tumorigenic tumor tissue is eliminated by the supervising organism systems. A set of procedural steps or platforms constitutes a new strategy for curing malignant neoplasms (for the Krebs-2 model).

The first platform of the strategy is the found treatment regimen using the cross-linking CP cytostatic. This regimen is linked to phases of the ICL repair cycle and enables induction of large-scale apoptotic destruction of committed tumor cells and synchronization of TISCs in the final treatment-sensitive phase of the cell cycle.

The second platform of the strategy is the phenomenon of internalization of extracellular DNA fragments from Krebs-2 TISCs. This internalization is combined and synergizes with CP. As a therapeutic DNA we use of a complex double-stranded DNA-based preparation. The preparation contains two components. It is introduced into the demarcation point of two phases (NER and homologous recombination) of the ICL repair process. One component of the preparation was experimentally demonstrated to eradicate TISCs from an ascites graft in the metronomic regimen 1−12 h after administration of the cytostatic. The second component − in the metronomic regimen 18−30 h after administration of the cytostatic. Finally, we found a regimen for a single injection of this preparation. Founding regimen led to consolidation of two vectors of the therapeutic effect on Krebs-2 TISCs. Therefore, the composition of preparation components affects simultaneously and immediately cells that occur at final stages of the NER repair phase and cells that enter the homologous recombination phase. Thus, the vast majority of TISCs simultaneously experience the therapeutic destructive effect [3]. However, evaluation of the cell cycle state after cytostatic injections and counting of the TISC amount for several (up to 12) days after onset of therapy indicated that not all TAMRA+-detected TISCs were eradicated during the first treatment stage. The TISC population survived after therapy was synchronized in the treatment-sensitive G1/S phase of the cell cycle.

The third platform of the strategy is as follows. The obtained result enabled the final regimen of treatment with the cytostatic and double-stranded DNA preparation. The final treatment is performed on the day when most tumor cells are destroyed by the cytostatic and DNA preparation. TISCs and residual differentiated tumor cells are synchronously accumulated in the G1/S phase sensitive to therapy. This day is determined individually for each experiment and varies (for Krebs-2) within 8−12 days since the first cytostatic injection [5].

Therefore, we developed a strategy to affect TISCs, which we called the 3+1 strategy. Therapy based on the new strategy resulted in complete cure from experimental Krebs-2 ascites tumor in mice.

In this study, the developed strategy was applied to Krebs-2 solid tumor. In the experiments, we used both a developed tumor (0.1-0.5 cm^3^ in size) and a non-palpable graft. Several compositions were used as DNA preparations. In some cases, compositions were supplemented with a protamine protector. The experiments were performed using the same Krebs-2 strain in different seasons for two years (January 2016, June 2016, and April 2017). Therapy with a specified time profile of the ICL repair cycle [3, 5] was found to be effective only in the winter season (at the place of research, Novosibirsk, Russia). The results obtained so far indicate that this effect was associated with seasonal variability of the time profile of the ICL repair cycle.

## RESULTS

### Efficacy of 3+1 winter 2016 therapy

**Figure 1:**
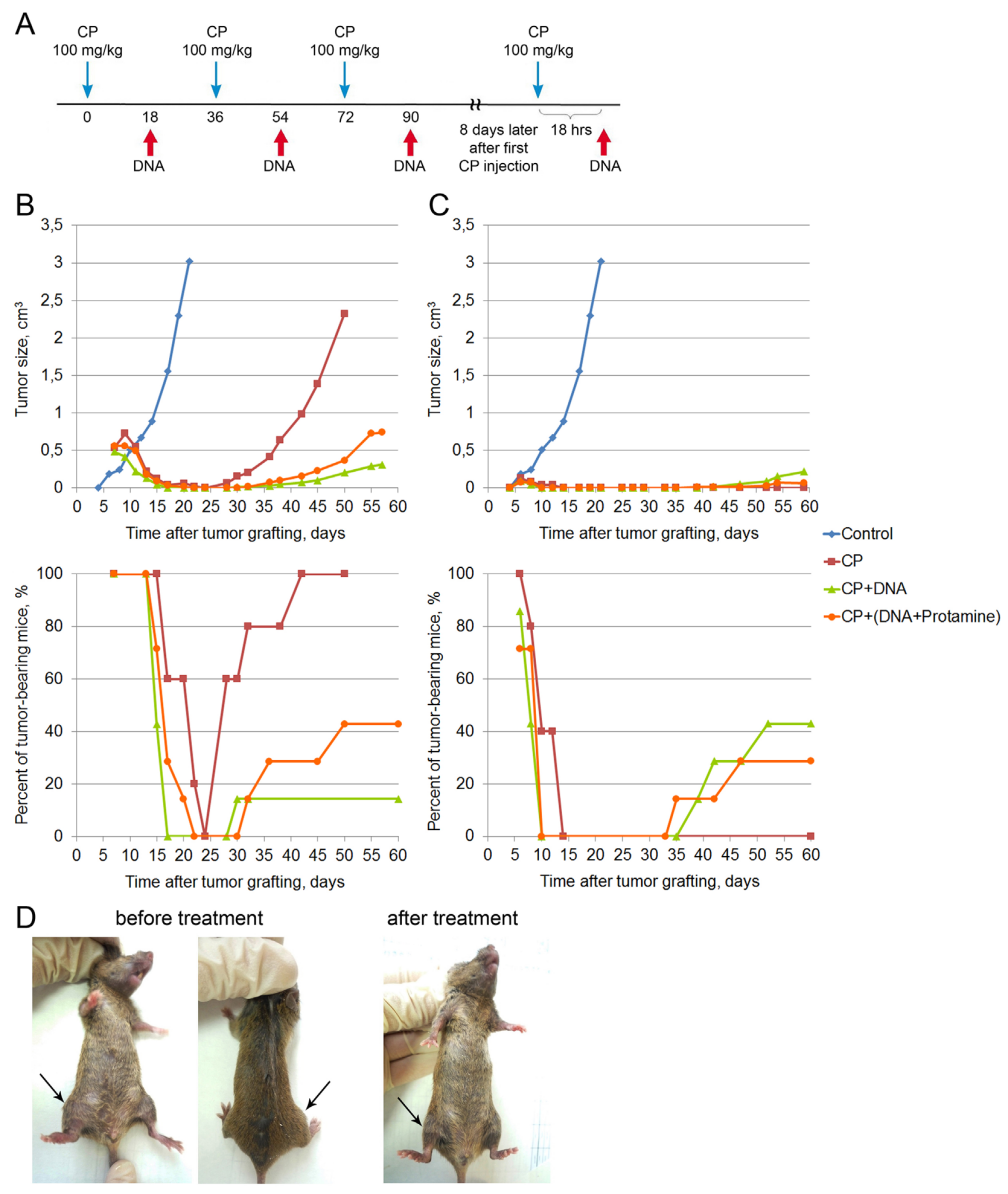
Treatment of a Krebs-2 solid tumor (winter 2016) **(A)** A schedule of injections. Blue arrow corresponds to CP injection, DNA injections are denoted by a red arrow on the schematic. DNA preparation was a mixture of equal amounts of native human DNA and salmon DNA (3 : 5 = cross-linked : native). **(B)** Dynamics of changes in the tumor size and number of animals with tumor upon treatment of a 0.5 cm^3^ graft. Data for the following animal groups treated according to the schedule (A) are shown: control, untreated, CP, CP-only; CP+DNA, CP and DNA injected directly into the tumor at a dose of 1 mg/mouse (first injection of 0.5 mg), CP+(DNA+protamine), CP and DNA injections in combination with protamine given intravenously at a dose of 0.1 mg. **(C)** Dynamics of changes in the tumor size and number of tumor-bearing animals upon treatment of a non-palpable graft. Treatment groups are the same as in (В). **(D)** An animal with an initial tumor size of 0.5 cm^3^ (before treatment) and also the animal with complete cure 210 days after the start of treatment (after treatment).

Having obtained a positive result in curing mouse Krebs-2 ascites tumor [5], we performed a series of experiments on curing a solid Krebs-2 graft. In January 2016, mice were intramuscularly inoculated with 300,000 Krebs-2 ascites cells. Seven days after the inoculation, when the tumor reached ∼0.3-0.5 cm^3^ in size, mice were treated according to the new 3+1 strategy. Mice received three CP injections at a dose of 100 mg/kg of animal weight at 36 hour intervals (arrest of three repair cycles) and a DNAmix injection 18 hours after each cytostatic injection (eradication of the main fraction of Krebs-2 TISCs) (Figure 1A). The DNAmix preparation was injected directly into the tumor or intravenously after formation of the complex with protamine. On day 8 after the first CP injection, animals received additional injections of the cytostatic and, 18 hours later, DNA preparation at similar doses (eradication of the residual population of Krebs-2 TISCs).

The data on tumor growth and the number of animals with tumor are shown in Figure 1B. Top panel of the Figure 1B summarizes the data on the average tumor size in each animal group. In control animals, rapid tumor growth is observed, and by day 20 following tumor cell injection the tumors reach the volume of 3 cm^3^. In the treated mice, tumor regression or complete disappearance is observed by day 4 following the treatment (day 11 after tumor cell injection). Tumors relapse around day 30. Figure 1B (bottom panel) shows the percentage of tumor-bearing animals across the experiments. Remission is restricted to the period from day 17 to day 30 following tumor engraftment (duration of remission differs between the groups). CP-treated animals had the shortest remission time, whereas CP+DNA–treated animals (intratumoral injection) remained relapse-free the longest. In this group, only 1 mouse out of 7 had a tumor at day 85 after tumor engraftment.

CP-treated animals had large tumors and were euthanized on day 50 for ethical reasons. In the CP+DNA+protamine group, during the third month the animals either succumbed to disease relapse (43%) or to systemic inflammatory reaction and multiple organ failure due to massive tumor lysis (57%) [9, 10]. As for the CP+DNA group, one mouse died of the tumor progression (14%), two mice (29%) dies of the tumor lysis-induced inflammatory syndrome, with 57% of mice remaining tumor-free up to the day 210. Representative images of mice before and after the treatment (day 210) are shown in Figure 1D.

In parallel, the same experiment was repeated, however the design was slightly modified: CP and DNA injection was started 2 days after tumor inoculation (Figure 1C). In other words, the treatment began when the tumor had engrafted, but was not yet palpable. We expected to see more pronounced effect of tumor growth suppression. Indeed, by the day 6 following tumor cell injection, 71-100% animals had tumors (Figure 1C, bottom panel), and by day 10, the signs of the graft disappeared in the groups that were administered CP+DNA in any form. No tumor was detected in any animal of the CP+DNA or CP+DNA+protamine groups until day 35-39. In the CP group, the tumor was not palpable in any animal from day 14 to day 75 of the experiment.

Animals that were given CP+DNA died during the days 70-85: 28-43% of the mice had tumor relapse, with the rest of the animals dying from tumor lysis syndrome. In the “CP” group all mice died without tumor signs from day 75 to day 110.

Thus, under this experimental setting (non-palpable grafts), CP monotherapy had stronger antitumor effect than the combined CP+DNA (intratumoral injection) or CP+DNA+protamine (intravenous injection) therapies.

### Efficacy of 3 + 1 summer 2016 therapy

**Figure 2:**
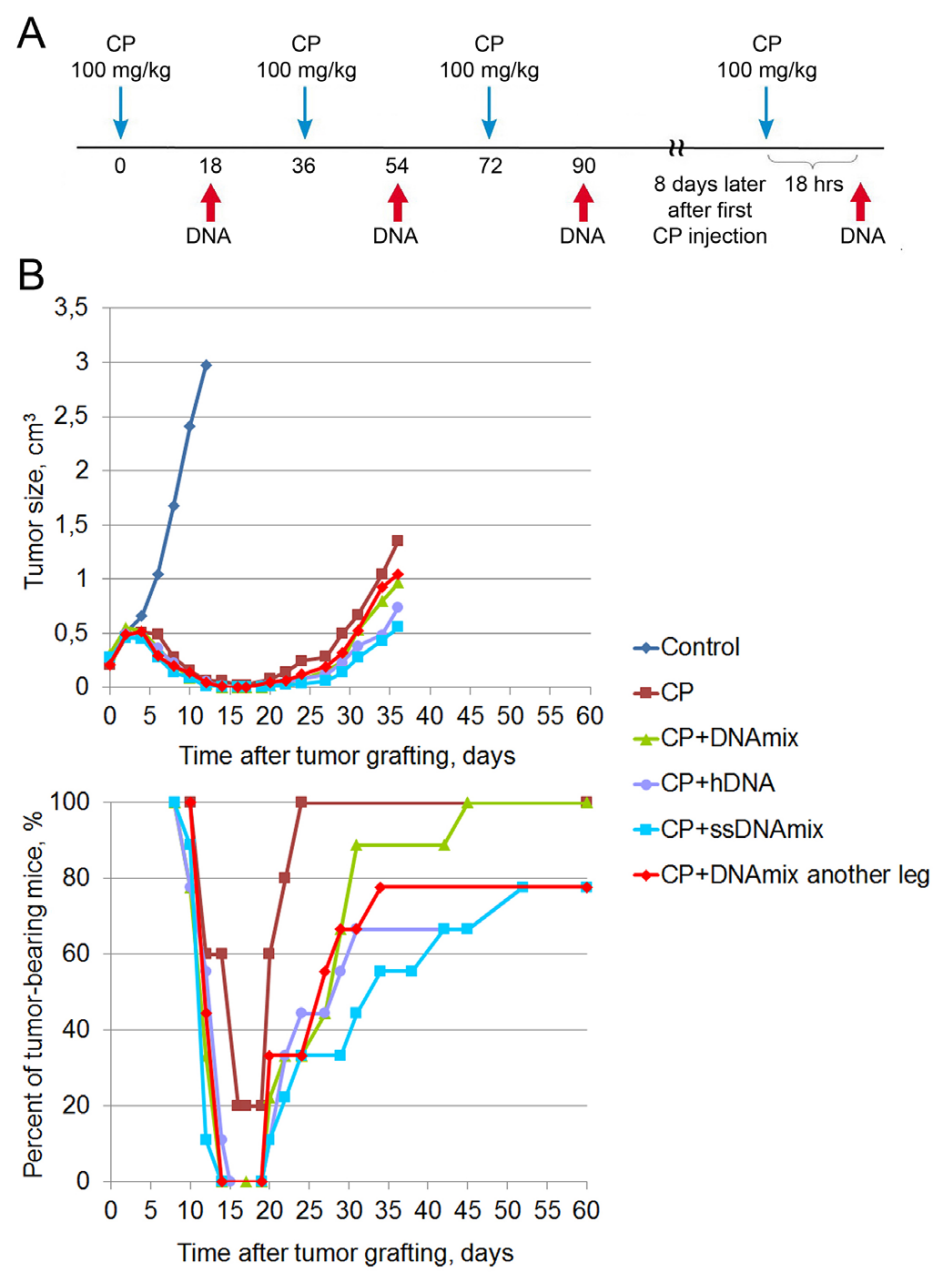
Treatment of a Krebs-2 solid tumor (summer 2016) **(A)** A schedule of injections. **(B)** Dynamics of changes in the tumor size and number of animals with the tumor. Data for the following animal groups treated according to the schedule (A) are shown: control, untreated; CP, CP-only; CP+DNAmix, intratumoral injections of CP and DNA; DNA preparation was a mixture of equal amounts of native human DNA and salmon DNA (3 : 5 = cross-linked : native). CP+hDNA, injections of CP and human DNA; CP+ssDNAmix, injections of CP and salmon sperm DNA (3 : 5 = cross-linked : native). CP+DNAmix another leg, injections of CP and DNAmix in the contralateral tumor-free leg.

After conducting the first two experiments, we decided to perform a third experiment, which was supposed to give a definitive answer to the question of the efficacy of using the CP cytostatic alone according to the “3+1” regimen. In June 2016, mice were intramuscularly inoculated with 400,000 Krebs-2 ascites cells. Eight days after inoculation, when the tumor reached ∼0.3-0.5 cm^3^ in size, mice underwent treatment according to the developed regimen (Figure 2A). The following groups were used: CP, CP+DNAmix, CP+hDNA, CP+ssDNAmix, and CP+DNAmix into another leg.

Tumor growth rates in different treatment groups are summarized in Figure 2B. Lower panel of the Figure 2B shows the percentage of tumor-bearing animals. During the period from day 14 to day 19 after tumor cell engraftment, complete remission is observed, which lasts differently for different treatment groups. In the CP group, remission period was the shortest (4 days) and was achieved in only 80% of animals. By the day 60, 78-100% of mice across all treatment groups had tumor relapse. For ethical reasons, mice were euthanized once the tumors became too large. Thus, the data from this experimental series appear to conflict with the results of the two first experiments.

### Revision of ideological platforms of the 3+1 strategy in the summer season

As already mentioned, three ideological platforms form the essence of the developed strategy. The first is a temporal profile of the repair cycle of CP-induced ICLs. The second is a new composite DNA-based preparation and a calendar of its administration related to the repair cycle configuration. The third is the found day for eradication treatment using the therapeutic regimen when the survived TISCs are synchronized in the sensitive phase of the repair cycle. The completely negative result of the last experiment based on the 3+1 therapy strategy indicated that parameters of the main ideological platforms underwent a fundamental change. For this reason, we revised all basic characteristics of both TISCs internalizing the TAMRA DNA probe and the entire population of tumor cells.

**Figure 3:**
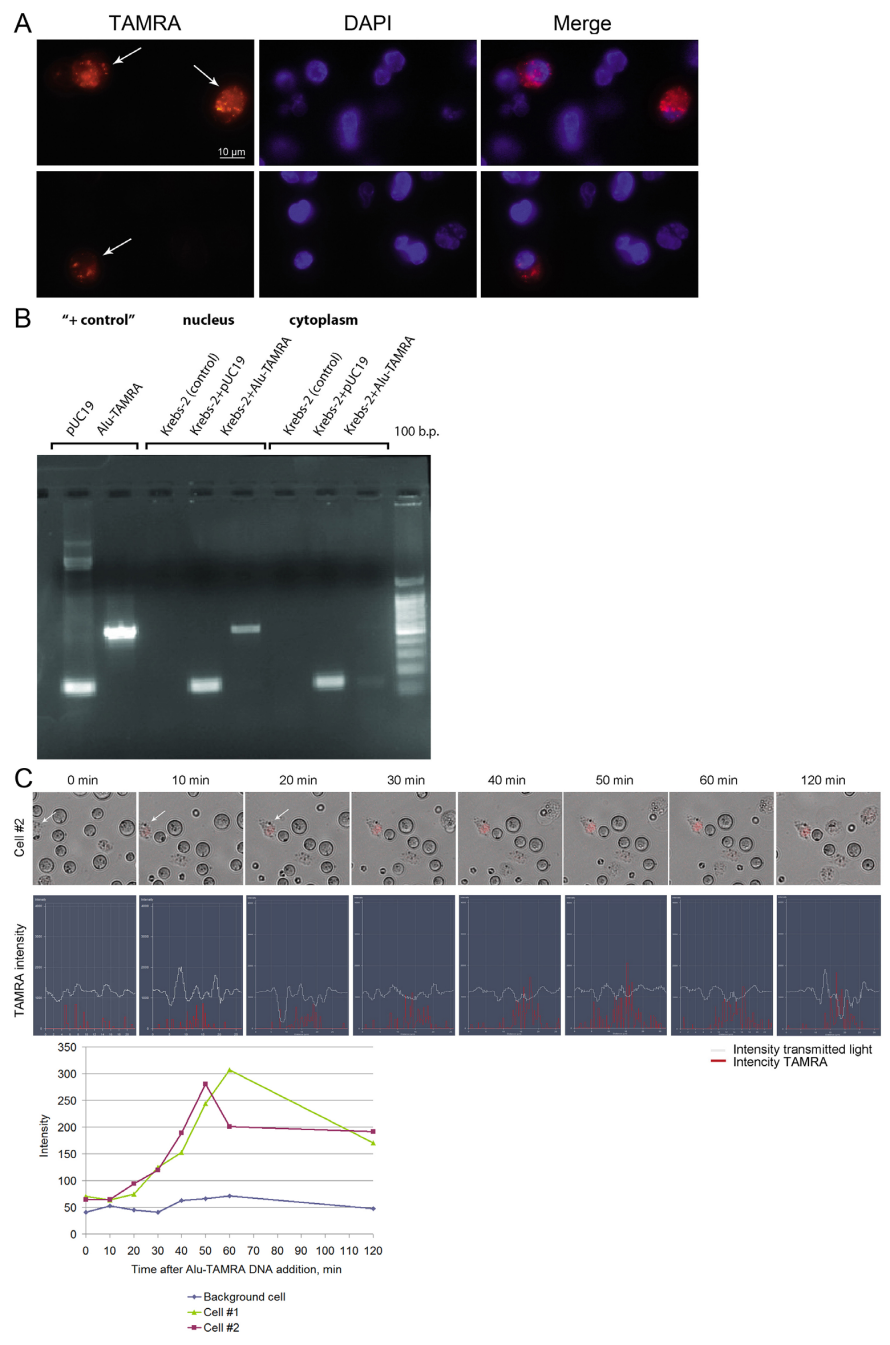
Internalization of a TAMRA DNA probe into Krebs-2 tumor cells **(A)** Fluorescence microscopy of Krebs-2 tumor cells incubated with TAMRA DNA probe for 1 h. Two representative fields are shown, with TAMRA+ cells indicated by the arrows. Cells were imaged in TAMRA and DAPI channels, merged image is shown. **(B)** Electrophoretic analysis of PCR amplification products of internalized DNA material distributed over the main cellular compartments. Krebs-2 tumor cells were incubated with plasmid pUC19 DNA or Alu-TAMRA DNA probe for 1 h, the cells were treated with DNase and proteinase K. DNA isolated from cytoplasm and nucleus fractions was used in PCR with appropriate primers. Krebs-2 (control) - DNA from intact Krebs-2 cells. “+ control” - PCR products with pUC19 DNA or Alu-TAMRA DNA as a template. Following the incubation of Krebs-2 cells with pUC19 DNA or Alu-TAMRA DNA probes, the PCR products become detectable in the nucleus and the cytoplasm. **(C)** Confocal analysis of the dynamics of labeled material accumulation in Krebs-2 cells from 0 to 120 min. TAMRA signal intensity profiles across two individual cells.

We found that the percentage of cells internalizing the fragments of double-stranded DNA did not change for at least five years of observation and ranged 0.5-7% [3, 5, 6]. The dynamics of internalization and the distribution of internalized DNA over the main cell compartments (nucleus and cytoplasm) also remained unchanged (Figure 3). Over the years, the efficiency of DNA uptake was measured multiple times. Depending on the approaches used (number of E. coli colonies formed upon transformation of the internalized plasmid DNA, or qPCR) the numbers varied from 0,25 to 1,66% of the haploid genome size [6 and unpublished data].

**Figure 4:**
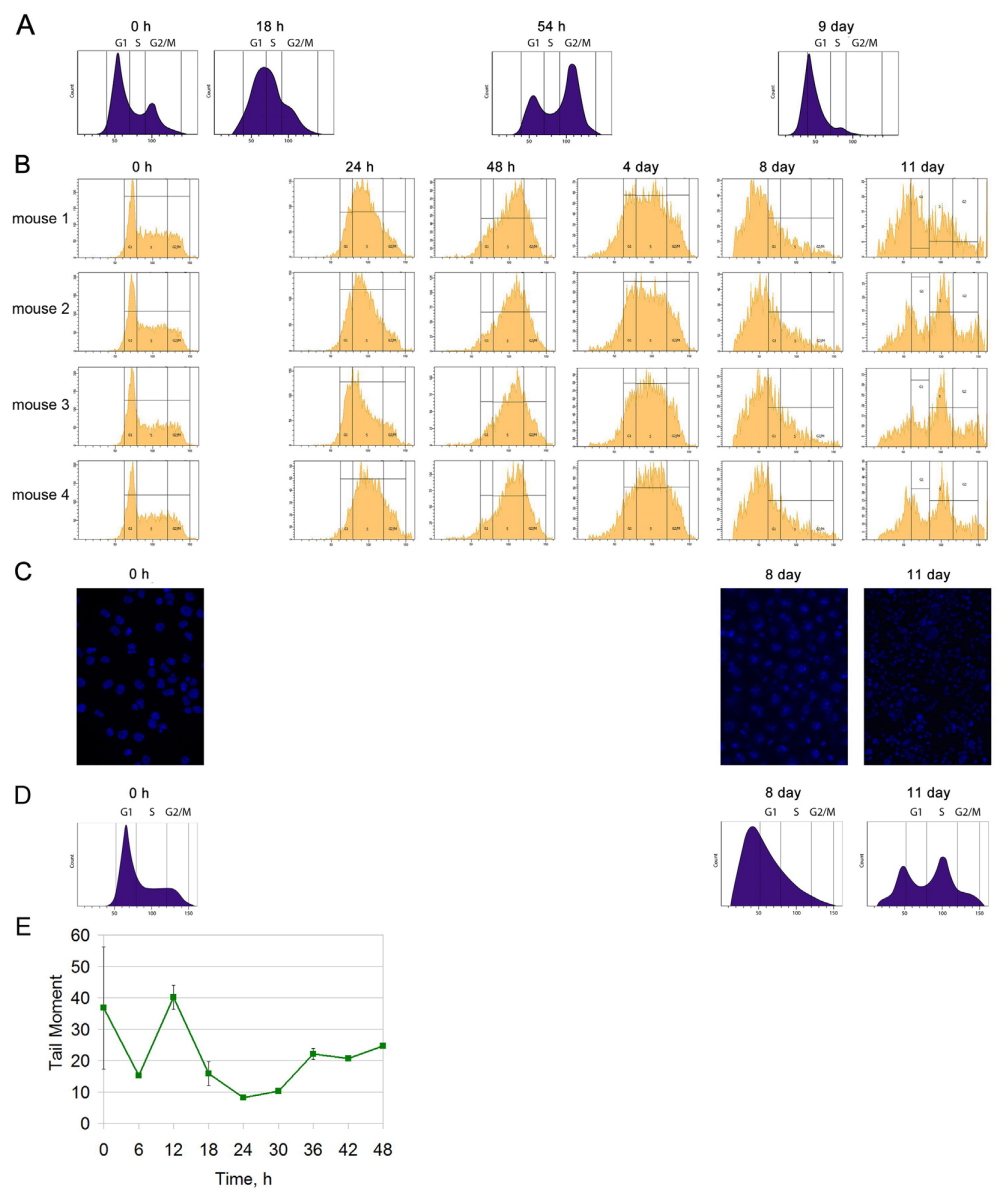
Cell cycle analysis of Krebs-2 ascites **(A)** Schematic results according to [5]. **(B)** Cell cycle analysis of Krebs-2 ascites for 4 mice after three-fold treatment with the CP cytostatic (100 mg/kg) at 36 hour intervals. **(C)** Visual condition of Krebs-2 ascites cells (DAPI) after three-fold CP injections. **(D)** A schematic of the results from (B). **(E)** The repair cycle of ascites cells; the mean values and standard deviations are given for 3 mice.

We analyzed the cell cycle of Krebs-2 ascites cells. Also we evaluated the amount of cells capturing TAMRA-labeled DNA after three-fold treatment with CP (100 mg/kg every 36 h) using a regimen similar to that presented in a study [5]. In the summer of 2016, four mice with grafted Krebs-2 ascites received three injections of the CP cytostatic at a dose of 100 mg/kg (each 36 h). Beginning from day 0, ascites cells were taken from animals every day for analysis of the cell cycle (Figure 4B).

No synchronous exit of the Krebs-2 cell population to the sensitive G1/S phase was found, as it was demonstrated in the winter experiments [5]. On day 8, there was some synchronization in apoptotic degradation of the main fraction of tumor cells, which did not end by synchronization in G1/S. On day 11, the Krebs-2 cell population demonstrated an unsynchronized cell distribution over the synthetic phase and the exit to mitosis. Also, no TAMRA+ cell accumulation was detected after treatment with CP on days 8−11, as it was shown [5] (Figure 4A and 4D). An evaluated amount of TAMRA+ cells on days 0 and 8 after treatment onset is presented in Table 1.

**Table 1:** Analysis of the amount of TAMRA+ cells in ascites after three-fold injections of CP or CP+DNA on days 0 and 8 after the first CP injection

	CP				CP+DNA	
	Mouse 1	Mouse 2	Mouse 3	Mouse 4	Mouse 1	Mouse 2
Day 0	0.62%	0.3%	0.12%	0.54%	0.35%	0.39%
Day 8	0.46%	0.81%	0.12%	0.39%	0.19%	Several cells

In parallel with this, animals with Krebs-2 ascites were injected with CP and the DNAmix preparation according to the developed regimen. It was intended to assess if there were TISCs that did not capture DNA after three-fold (CP+DNA) treatment. It was found that cells capable of capturing labeled DNA were detected on day 8 after the treatment (Table 1). Thus, three-fold treatment of Krebs-2 ascites with CP+DNA every 36 hours in the summer period did not completely eliminate TAMRA positive Krebs-2 stem cells from ascites, as it was shown for the winter period in the study [5].

Thus, there remained the only factor that might underlie the observed changes in basic platforms of the 3+1 strategy. It was the time profile of the ICL repair cycle. In September 2016, we revised of the repair cycle of CP-induced ICLs. The temporal profile of repair was found to have a different configuration as compared to that in the winter period. Accumulation and repair of double-strand breaks occurred sequentially, without a latent phase. The entire repair process was completed in 24 h (Figure 4E).

**Figure 5:**
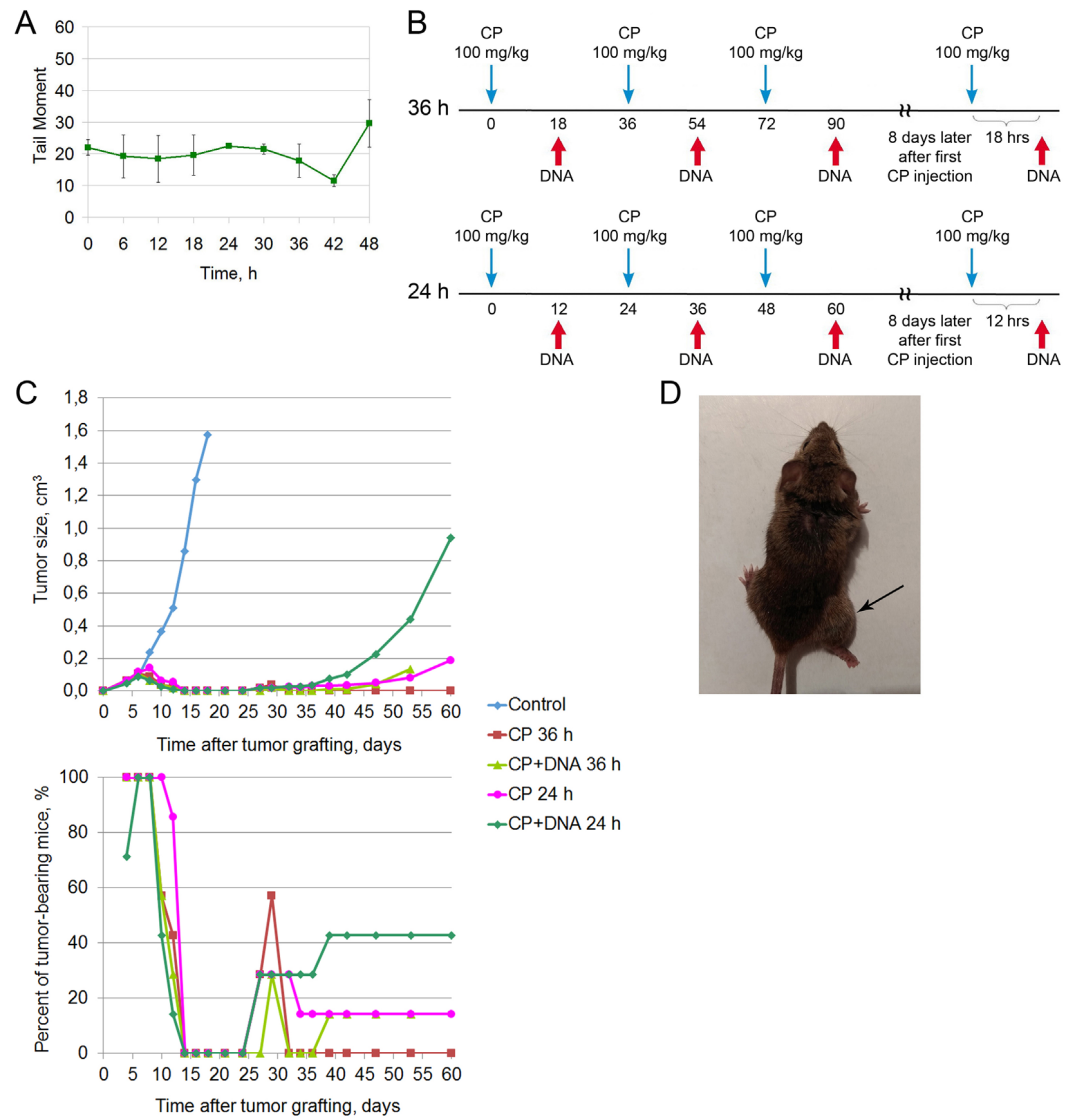
Treatment of a Krebs-2 solid tumor (spring 2017) **(A)** The repair cycle of ascites cells; the mean values and standard deviations are given for 2 mice. **(B)** A schedule of injections for 36 and 24 hour intervals of CP administration. **(C)** Dynamics of changes in the tumor size, number of animals with tumor, and number of surviving mice. Data for the following animal groups treated according to the schedule (B) are shown: control, untreated; CP 36h and CP 24h, CP injections at 36 or 24h intervals; CP+DNA 36h and 24 h, co-injections of CP and DNA preparation at 36 or 24 h intervals. **(D)** Image of a mouse from the CP 36 h group, which relapsed on day 85 after inoculation.

The conducted experiments supposed that the main factor causing catastrophic changes in the biology of Krebs-2 cancer cells was the time of experiments, namely the winter and summer seasons. In this regard, in the spring 2017, we repeated repair cycle experiments and biological tests for graft development on solid Krebs-2 tumor. The results of these experiments are presented in Figure 5.

The ICL repair cycle was found to alter again the temporal profile (Figure 5A). The lag period reappeared. Сomplete repair of double-strand breaks shifted 6 hours later the CP injection compared to the time reported in a study [3] and occurred at 42 hours. In the conducted experiments, there was no clear boundary between the 0 timepoint and the 6−12 hour timepoint. This fact is supposed to be associated with that the tumor contained an increased number of transient double-strand breaks during experiments, which resulted in overestimation of the initial index. The processes of break filling and the appearance of true double-strand breaks associated with initiation of NER overlapped. This distorted the clear picture of NER initiation. Despite the differences between the new repair cycle and the cycle underlying therapy, we decided not to change the administration regimen of CP and DNA preparation. The rationale behind this choice (for CP injections) was that 36th hour occurred in the time interval spanning the homologous recombination phase and the end of the repair cycle. Notably, DNA administration at 18 hours still occurred in the lag phase between accumulation of double-strand breaks and the onset of their disappearance. That is, in general, the overall picture of injections does not contradict the logic of selected regimens [5].

To evaluate the therapeutic effect of DNA preparation in the final phase of double-strand break accumulation, the experimental design was supplemented with an additional regimen. In it, CP and DNA were administrated according to the 3+1 regimen with a 12 hour interval of the DNA injection after the CP injection. In this case, CP was injected each 24 hours.

In April 2017, mice were intramuscularly inoculated with 1,000,000 Krebs-2 cells. On day 4 (tumor size ≤ 0.1 cm^3^), cytoreducive therapy was initiated (Figure 5B). There were 2 intervals of CP administration: the standard 36 hour interval and an additional 24 hour interval. The DNA preparation was introduced 18 and 12 hours, respectively, after CP.

The dynamics of the graft development looks as follows (Figure 5C). During the days 14 to 24 following tumor engraftment, the complete disappearance of the tumor was observed in all mice from all groups. Some mice then relapsed and progressed (as in CP+DNA24 h group) or the graft entirely disappeared (as in CP 36 h group, Figure 5C, lower panel). This experiment demonstrated that the regimen CP 36h was the most potent, as no relapses were detected on day 60, and only a single mouse relapsed on day 85 (Figure 5D).

Mice were either euthanized once their tumors became too large (14-43% of animals in different groups), or succumbed to the systemic inflammatory response and multiple organ failure [9, 10]. By day 210, 57% animals were alive and tumor-free in the CP 36 h group, whereas 43% of such mice remained in the CP 24 h group. Inclusion of DNA into the therapy resulted in fewer cured mice, as compared to the CP-only regimen. By day 210, the groups CP+DNA 36h and CP+DNA 24h encompassed 29% and 14% tumor-free animals, respectively.

It is possible that DNA preparation has buffered the CP activity due to slightly altered repair profile, as was observed previously [3]. Thus, overall our results are consistent with the data obtained in the first experimental series (performed in winter) and confirm the efficacy of CP as a monotherapy (36 h interval), when it matches the tumor cell repair cycle.

## DISCUSSION

### Curing mice from a developed solid Krebs-2 cancer graft

We found a therapeutic regimen to cure mice from solid Krebs-2 tumor, which was the first major finding of this study. The main parameters of this regimen correspond to the regimen of curing Krebs-2 ascites tumor [5]. The size of a solid lesion is apparently important for effective therapy. In all experiments with a tumor of more than 1 cm^3^, eradication of the graft failed (data not shown). This is presumably due to the fact that the injected DNA preparation and, probably, CP do not reach target cells in a space occupying lesion. Tumor analysis using a thermal imager demonstrated that a Krebs-2 solid tumor lacked vascularization (data not shown). Therefore, both preparations should reach the target cells to effectively affect the tumor.

In the present study, we focused on both the synergistic effect of the cytostatic and DNA preparation and the effect of the cytostatic as a monotherapy in the found regimen of TISC synchronization. The CP cytostatic alone was experimentally found to have a pronounced therapeutic effect, effectively preventing the development of relapse after therapy. The cytostatic, as in treatment of the ascites form of Krebs-2 tumor, was used in the regimen of “an arrest of three repair cycles” and a final terminal injection at the time of synchronization and accumulation of TAMRA+ TISCs. Despite the fact that in the first experiments (winter 2016) relapses developed in all mice in groups, they emerged on the 80th day after treatment. Given that control mice died on days 14−18 after inoculation, this delay in the relapse development indicated a significant therapeutic effect of the cytostatic alone when it was administered in accordance with the described regimen.

In the second series of experiments (spring 2017), CP as a monotherapy effectively reduced a developing graft and prevented the development of relapses. This difference in the effect, a prolonged delay in winter 2016/complete remission in spring 2017, is likely related to two reasons. One option is the CP quality or changes in the biological tumor features manifesting in the variability of the temporal profile of the ICL repair cycle in different periods of the year. “The effect of a different manufacturer” was observed by us during the second phase of clinical trials of the preparation Panagen. Namely, mobilization time of hematopoietic progenitors differed by 4 days, depending on a clinical trial center and the cytostatic used according to the FAC or AC regimens [11]. It is possible that complete remission in the graft development is associated with the use of a standard regimen of action when the repair cycle duration is extended to 42 hours. If so, further research in this direction is required because this finding suggests the opportunity for rapid introduction of the found conditions into the clinical practice. Therefore, we discovered second major finding of this study. It is the therapeutic effect of the CP cytostatic as a monopreparation related to the temporal profile of the repair cycle of cytostatic-induced ICLs.

### Temporal profile seasonality of the ICL repair cycle in Krebs-2 tumor cells

**Figure 6:**
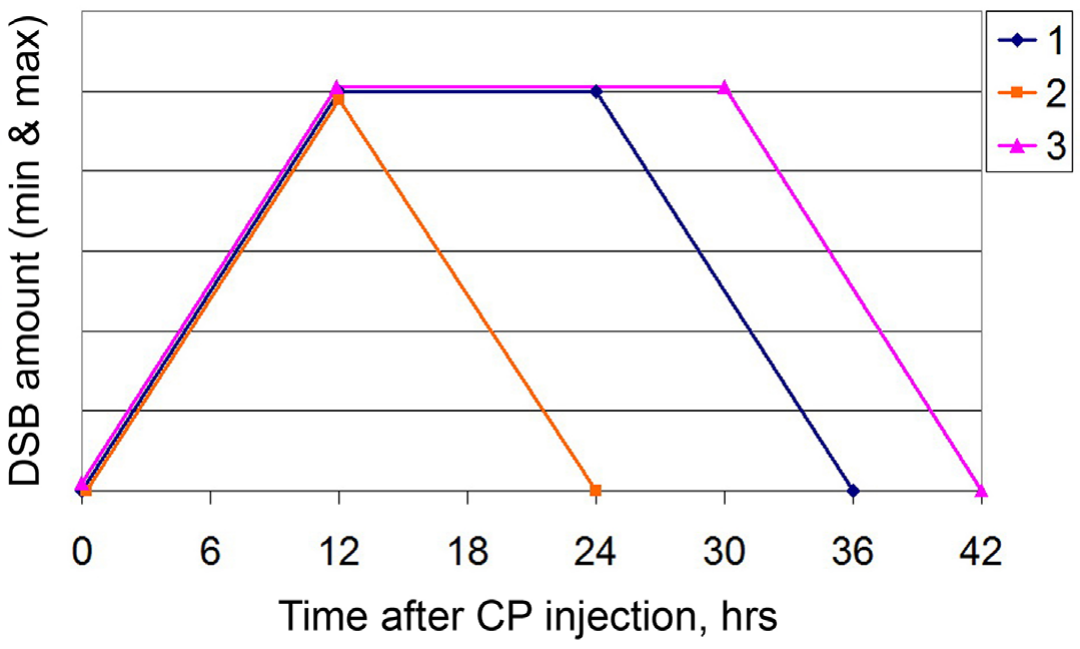
A schematic of the DSB dynamics 1: as in the study [3]; 2: in the summer of 2016; 3: in the spring of 2017. For the sake of visualization simplicity, the plot is based on the minimum and maximum values of DSBs that were detected in independent experiments, regardless of the detection method. Thus, the Y axis shows the arbitrary scale normalized to the minimum and maximum values.

In this study, we discovered for the first time the phenomenon of seasonal changes in the ICL repair cycle in Krebs-2 tumor cells (Figure 6). Three consecutive experiments on curing a solid Krebs-2 graft revealed that the third, in succession and timing, experiment gave absolutely different results compared to those of the first two. In the third experiment, similar therapeutic procedures failed both to stop the development of a grafted tumor and to prevent relapse in any of the groups. Revision of all platforms underlying the ideology of the 3+1 strategy has demonstrated that a change in the parameters of these platforms is associated with a categorical change in the temporal profile of the repair cycle of CP-induced ICLs. The repair cycle reduced in time, and the latent phase interval between accumulation of double-strand breaks and their repair disappeared. The main difference between the third experiment and the first two was that it was performed in the Siberian summer season. All additional experiments on revision of other platforms of the strategy were also carried out in summer.

Studies verifying the idea, which were carried out in the next winter season of 2017, demonstrated the complete restoration of the positive results obtained in the first two experiments on curing experimental mice from a Krebs-2 solid graft. The basis for this change was again a change in the repair cycle and its return, in general, to the initial temporal profile. Therefore, the third major finding of this study was detection of the seasonal variability in the response of a cancer cell or tumor, in general, to the effect of cytoreductive therapy. Despite the fact that the treatment of tumor cells leads to large-scale apoptotic destruction of committed cancer cells (Figure 3), TAMRA positive TISCs do not synchronize in the sensitive phase of the cell cycle and remain unaffected by therapy (Figure 4). It is noteworthy that the ssDNAmix composition was most effective in the summer experiment. This fact is in line with the previously stated idea that different double-stranded DNA-based preparations have different mechanisms of interference with two repair mechanisms, NER, and homologous recombination [3]. In the summer, the repair cycle in Krebs-2 cells is shortened. Then, ssDNAmix injections 18 hours after the CP injection occur during the ongoing homologous recombination. It was this composition applied in this interval of the repair process that had a high therapeutic effect, effectively inhibiting or stopping the development of a tumor graft [3]. This observation is an additional fact confirming the observed changes in the temporal profile of the repair cycle.

Seasonal and circadian changes in biological systems are well known and characterized [12–15]. Seasonal changes in expression of microRNA genes [16, 17] and production of the tumor necrosis factor [18] have been reported. In our early experiments, we demonstrated seasonal activity of synergistic action of CP and human native DNA-based preparation on stem hematopoietic cells. In the winter period, the synergistic action resulted in knock-out of the lymphoid hematopoietic lineage and formation of the time interval, which we called “death window”, that disappeared in summer [9]. There is a seasonal correlation between the incidence rate of primary cancers, which is associated with the amount of produced vitamin D [19], and the rate of postoperative pulmonary metastases in breast cancer [20]. Many biochemical parameters of breast cancer are known to have seasonal variability [21]. There are also numerous experimental studies characterizing seasonal and circadian changes in the proliferative activity of various cell types, including stem cells [22–28].

Thus, the process of chromatin repair is directly related to cell proliferation. It may be assumed that, for Krebs-2, the observed seasonal variability of the repair cycle profile is a consequence of altered temporal profile of proliferation.

In conclusion, we note that one of the possible and very likely reasons for the tremendous differences between the treatment efficacies observed in our experiments is the seasonal cyclicity in the temporal profile of the interstrand DNA crosslink repair. Further studies performed during multiple seasons are needed to statistically confirm whether this is indeed the case.

## MATERIALS AND METHODS

We used 2-6-month old CBA/Lac mice strain (males and females, 18-30 g) bred at the Institute of Cytology and Genetics, SB RAS. Animals were grown in groups of 5-10 mice per cage with free access to food and water. All experiments were performed in accordance with the protocols approved by the Animal Care and Use Committee of the Institute of Cytology and Genetics.

Krebs 2 ascitic carcinoma is a strain-nonspecific tumor derived from epithelial cells; all inbred mouse strains can be challenged with Krebs 2 tumor cells. When challenged subcutaneously or intramuscularly, it grows as solid nodes. It is weakly immunogenic for mice of all strains. It does not give rise to metastases [29–32].

The complex composite DNA preparation (DNAmix^®^) was a mixture of equal amounts of native human DNA and salmon DNA (3 : 5 = cross-linked : native); in some cases, the DNA was stabilized by the protein protamine (1 to 0.8 by the amount). The preparation was prepared as described in a study [10].

TAMRA labeling of DNA probes and delivery of human TAMRA-labeled Alu DNA into Krebs-2 ascites cells was performed as described in a study [3].

Analysis of DSB formation in Krebs-2 ascites cells and a comet assay to quantify DSB-positive cells were also performed as described in the study [3].

Cell cycle analysis of Krebs-2 ascites cells was performed as described in a study [5].

### Regimens of preparation injection in mice

#### General comments

Identification of the exact timing of interstrand cross-link repair is central to our approach, and this was followed by tracking the appearance, peaking and drop to the background value of the number of DSBs. When using the conventional DSB-repair assay using γH2X-specific antibodies, the data could not be retrieved for the last phase of DSB repair, even though this method is known to perform well for cell lines in both FACS-based assays and when repair foci are counted under the microscope [33, 34]. 48 h time-course analysis of cell samples taken from the peritoneal ascites of CP-treated animals was nearly impossible to interpret, as this was accompanied with a strong non-specific fluorescence associated with preapoptotic mitochondrial activity. Thus, whether DSB repair was completed could not be reliably monitored by FACS or microscopy analysis. This technical obstacle prompted the use of an alternative assay to measure DSB repair, namely the comet assay. This approach was robust in identifying the exact time of when the repair has been completed (visualized as the disappearance or strong reduction of the comet tails).

The following set of standard parameters was used to assess the therapy efficacy. Tumor relapse and time of tumor relapse, development of the tumor lysis syndrome [9, 10], relapse-free survival of mice beyond day 90 from the therapy beginning, death of mice due to multiple organ failure, death of mice due to tumor relapse [5]. Body weight dynamics is generally known as an important parameter informing on the efficacy of the therapeutic intervention. Nonetheless, in our specific experiments, this parameter turned out to be poorly informative and highly individualized. Throughout the experiments, differences in body weight values were either below statistical significance or indicated nothing more than the weight fluctuated in time (Supplementary Figure 1) [35]. Due to this obstacle and based on our earlier observations, we chose to adopt the following weight-based approach to monitor the treatment efficacy. We noted, that the strongest tumor-killing effect was observed in remission and upon resorption of the primary transplant, - which was accompanied with the loss of up to 30% of mouse weight. So, the animals were weighted at three time points: before the experiment, in the beginning of remission stage (days 10-15), and on days 20-25 (continued remission). Based on the mouse weight changes, the corrective therapy (intraperitoneal administration of 5% glucose and gentamicin at a dose 4 mg/kg of weight, soft food – dry standard food soaked in water) was given to the animals or not.

The numbers of tumor cells infused per animal varied from 300 000 to 1 000 000. For Krebs-2 mouse cancer, this number only affects the time needed for the graft to become palpable and varying the initial cell numbers was needed to achieve faster onset of the graft growth. Age differences, and consequently weight differences among animals in different experiments were based on the following. When treating ascites form of Krebs-2 [5, 10], we noted that heavier animals were more likely to survive the extreme exhaustion caused by the treatments. In the first round of experiments described in the present study, we expected the same effect to occur and chose to work with older and heavier mice. This turned out not to be the case, as treatment of the solid form of Krebs-2 tumor resulted in less pronounced systemic effects. So, we switched to the typical (age- and weight-wise) mice in the subsequent experiments.

For the sake of uniformity, the figures provide the data summarizing average tumor size and percentage of tumor-bearing animals up to the day 60. However, the animals were monitored for longer, as noted in the description of the experiments in “Resultsˮ.

#### 3+1 winter 2016 therapy

A graft was inoculated into the right hind leg, in a muscle above the knee joint. In January 2016, 6-month-old female CBA mice were inoculated with 300,000 cells of 7-day-old Krebs-2 ascites (in 100 μL of saline). Seven days after inoculation, when the tumor reached 0.5 cm^3^ in size, or after 2 days, when the tumor was not yet palpable, the therapy was begun. Mice received three CP injections at a dose of 100 mg/kg of animal weight at 36 hour intervals and DNAmix injections 18 hours after each cytostatic injection. One group of animals received only CP injections (n=5), another group (n=7) received CP and the DNA preparation directly into the tumor at 3−4 sites, 1 mg/mouse (the first injection was 0.5 mg). The third group of animals (n=7) received CP and was intravenously injected with 0.1 mg of the DNA preparation mixed with protamine (1 to 0.8 by the amount). On day 8 after the first CP injection, animals received additional injections of the cytostatic (100 mg/kg) and, 18 hours later, DNA preparation at similar doses. The control group (n=5) with an engrafted tumor received no injections.

#### 3+1 summer 2016 therapy

In June 2016, 4-month-old female CBA mice were intramuscularly inoculated with 400,000 Krebs-2 ascites cells (in 100 μL of saline). Eight days after the inoculation, when the tumor reached ∼0.5 cm^3^ in size, mice underwent therapy according to the developed regimen. Mice received three CP injections at a dose of 100 mg/kg of animal weight at 36 hour intervals and a DNA preparation injection 18 hours after each cytostatic injection. DNA was injected directly into the tumor at 3−4 sites at 1 mg/mouse. On day 8 after the first CP injection, animals received additional injections of the cytostatic (100 mg/kg) and, 18 hours later, DNA preparation at similar doses. The following groups were used: CP (n=5), CP+DNAmix, CP+hDNA, CP+ssDNAmix, CP+DNAmix in another leg (n=9). The control group (n=5) with an engrafted tumor received no injections.

#### 3+1 spring 2017 therapy

In April 2017, 2−2.5-month-old female CBA mice with weight of 19−20 g were intramuscularly inoculated with 1,000,000 Krebs-2 cells in the right leg. On day 4 (tumor size ≤ 0.1 cm^3^), cytoreductive therapy was initiated. Mice received three CP injections at a dose of 100 mg/kg of animal weight at a 24 or 36 hour interval. The DNAmix preparation at a dose of 1 mg/mouse was injected directly into the tumor, at 2−3 sites, 12 or 18 hours after the next cytostatic injection, respectively. Eight days after the first CP injection, animals received an additional injection of the cytostatic (100 mg/kg) and DNA preparation after 12 or 18 hours. The following groups were used: CP 24 h, CP+DNA 24 h, CP 36 h, and CP+DNA 36 h (n=7). The control group (n=5) with a grafted tumor received no injections.

## SUPPLEMENTARY MATERIALS FIGURE



## Abbreviations

TISCs: tumor initiating stem cells
ICLs: interstrand cross-links
CP: cyclophosphamide
