# Supplementary material for: Efficacy of a new cancer treatment strategy based on eradication of tumor-initiating stem cells in a mouse model of Krebs-2 solid adenocarcinoma
Source: Oncotarget. 2018 Jun 19;9(47):28486–99. doi: 10.18632/oncotarget.25503 (PMC6033367; doi:10.18632/oncotarget.25503)
Supplement: Supplementary file 1 [file oncotarget-09-28486-s001.pdf]

Efficacy of a new cancer treatment strategy based on eradication of tumor-initiating stem cells in a mouse model of Krebs-2 solid adenocarcinoma

SUPPLEMENTARY MATERIALS

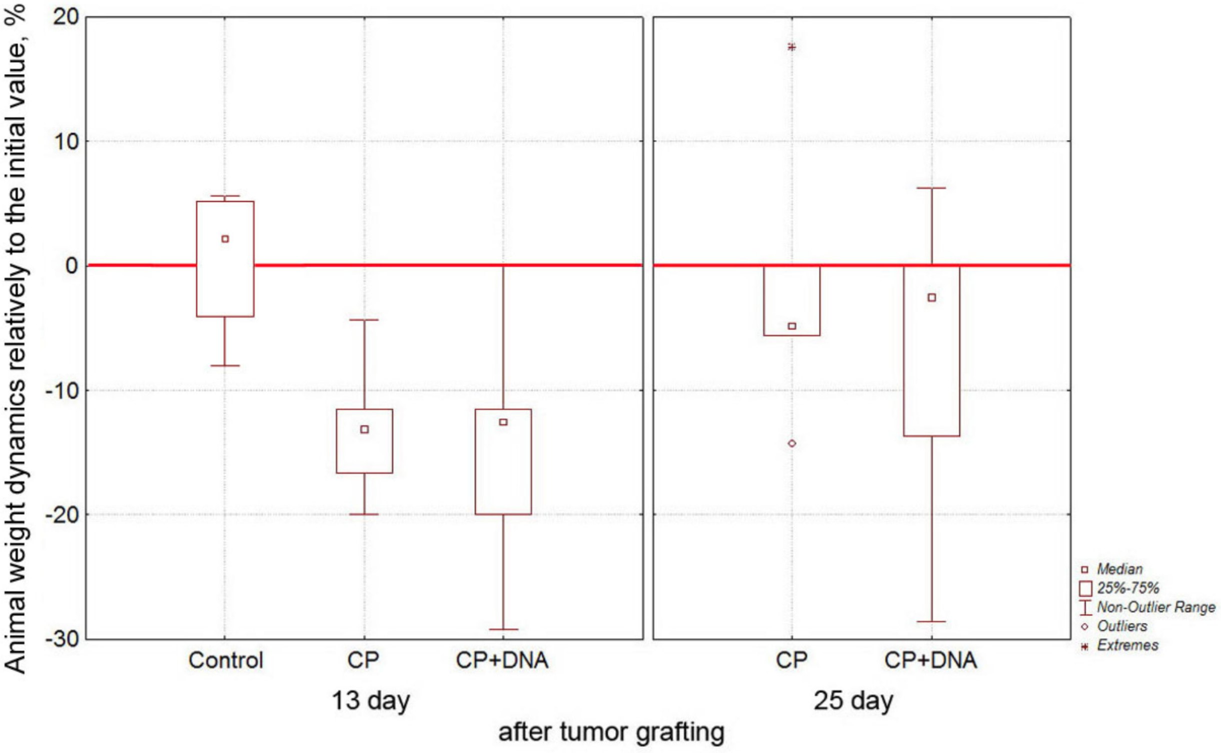

Supplementary Figure 1: Mouse weight changes relatively to the initial value. Positive values correspond to the weight gain, negative values indicate weight loss. Shown are the values normalized by the initial weight of mice for the control group (size-inferred value of the tumor weight on day 13 has been subtracted) and the mice administered with CP or CP+DNA on a 3+1 regimen on days 13 and 25 after tumor engraftment. Synergistic use of the two agents is the most destructive for the organism. On day 13 the animals may lose up to 30% of their initial (20-22g) weight.
